# A Semantic Approach to Describe Social and Economic Characteristics That Impact Health Outcomes (Social Determinants of Health): Ontology Development Study

**DOI:** 10.2196/52845

**Published:** 2024-03-13

**Authors:** Daniela Dally, Muhammad Amith, Rebecca L Mauldin, Latisha Thomas, Yifang Dang, Cui Tao

**Affiliations:** 1 The University of Texas Health Science Center at Houston School of Public Health The Brownsville Region Brownsville, TX United States; 2 Department of Biostatistics and Data Science University of Texas Medical Branch Galveston, TX United States; 3 Department of Internal Medicine University of Texas Medical Branch Galveton, TX United States; 4 School of Social Work The University of Texas at Arlington Arlington, TX United States; 5 School of Biomedical Informatics The University of Texas Health Science Center at Houston Houston, TX United States; 6 Department of Artificial Intelligence and Informatics Mayo Clinic Jacksonville, FL United States

**Keywords:** social determinants of health, ontology, semantics, knowledge representation

## Abstract

**Background:**

Social determinants of health (SDoH) have been described by the World Health Organization as the conditions in which individuals are born, live, work, and age. These conditions can be grouped into 3 interrelated levels known as macrolevel (societal), mesolevel (community), and microlevel (individual) determinants. The scope of SDoH expands beyond the biomedical level, and there remains a need to connect other areas such as economics, public policy, and social factors.

**Objective:**

Providing a computable artifact that can link health data to concepts involving the different levels of determinants may improve our understanding of the impact SDoH have on human populations. Modeling SDoH may help to reduce existing gaps in the literature through explicit links between the determinants and biological factors. This in turn can allow researchers and clinicians to make better sense of data and discover new knowledge through the use of semantic links.

**Methods:**

An experimental ontology was developed to represent knowledge of the social and economic characteristics of SDoH. Information from 27 literature sources was analyzed to gather concepts and encoded using Web Ontology Language, version 2 (OWL2) and Protégé. Four evaluators independently reviewed the ontology axioms using natural language translation. The analyses from the evaluations and selected terminologies from the Basic Formal Ontology were used to create a revised ontology with a broad spectrum of knowledge concepts ranging from the macrolevel to the microlevel determinants.

**Results:**

The literature search identified several topics of discussion for each determinant level. Publications for the macrolevel determinants centered around health policy, income inequality, welfare, and the environment. Articles relating to the mesolevel determinants discussed work, work conditions, psychosocial factors, socioeconomic position, outcomes, food, poverty, housing, and crime. Finally, sources found for the microlevel determinants examined gender, ethnicity, race, and behavior. Concepts were gathered from the literature and used to produce an ontology consisting of 383 classes, 109 object properties, and 748 logical axioms. A reasoning test revealed no inconsistent axioms.

**Conclusions:**

This ontology models heterogeneous social and economic concepts to represent aspects of SDoH. The scope of SDoH is expansive, and although the ontology is broad, it is still in its early stages. To our current understanding, this ontology represents the first attempt to concentrate on knowledge concepts that are currently not covered by existing ontologies. Future direction will include further expanding the ontology to link with other biomedical ontologies, including alignment for granular semantics.

## Introduction

### Background

Ontologies are an important resource that have advanced the biomedical sciences. Originating from the philosophical domain and later incorporated into the computing and information sciences, ontologies represent and model our physical reality using semantics to describe domain entities (ie, knowledge base) [1]. These artifacts can be used to house vocabularies to generate inferences with the help of software reasoners such as HermiT [2], ELK [3], and FaCT++ [4]. Logically structured vocabularies can be used with reasoning tools to implement problem-solving software in clinical settings. In addition, biomedical researchers have advanced and wielded ontologies to be used in applications for artificial intelligence, natural language processing, information retrieval, and indexing (eg, data integration, harmonization, and exchange) [5]. Some impactful examples of ontologies include the Systematized Nomenclature of Medicine–Clinical Terms [6] and Gene Ontology [7], which are hosted on the National Center for Biomedical Ontology [8] and the OBO Foundry [9]; for example, the National Center for Biomedical Ontology BioPortal is an open repository of >700 biomedical ontologies [8], whereas the OBO Foundry hosts interoperable biomedical and health ontologies that share a common framework [9]. All the OBO Foundry–approved ontologies are built upon the Basic Formal Ontology (BFO), a common upper-level ontology, for interoperability and reuse. More than ever, there is a strong need to use ontologies for social health behavior sciences with the downstream goal of harmonizing biological and behavioral data [10].

### Social Determinants of Health

Since the early 19th century, the public health community has sought to determine how social determinants are associated with behavior, health outcomes, and health inequalities [11]. Factors such as social position can influence an individual’s health status and thus lead to disease-inducing behaviors [11]. The link between social determinants and disease is a central point for public health research [11]. Over the years, public health researchers have classified these determinants as social determinants of health (SDoH). SDoH have been described by the World Health Organization as the conditions in which individuals are born, live, work, and age [12]. These nonbiological factors influence health outcomes in terms of health status, well-being, mortality, and life expectancy.

SDoH encompass many different areas, such as social and political context, governance, physical and living environment, community, safety, education, occupation, income, cultural and social values, biological and behavioral factors, wellness, food, and the health care system [12]. These categories can be represented by 3 levels of organization: macrolevel, mesolevel, and microlevel determinants [12]. Macrolevel determinants consist of socioeconomic hierarchies that govern access to resources in society through policy making [11]. Mesolevel determinants include concepts such as environment, neighborhood quality, occupation, and crime. This intermediate level is also concerned with psychosocial risk factors such as a stressful environment, the quality of social networks, and high physical or social demand [11]. Finally, microlevel determinants describe individual interactions, behaviors, lifestyle, and genetics [11]. Associated with these determinants are health inequalities, or the unfair and avoidable differences in health status among individuals [12], including inequities caused by structural or systemic factors.

### Research Objective

The overarching goal of this research was to develop a biomedical ontology to model and represent knowledge on SDoH. More specifically, this work attempted to provide a broad spectrum of concepts ranging from the macrolevel to microlevel determinants focusing on social and economic characteristics as well as social-related health policies. By developing an ontology for SDoH, we can standardize the current scientific knowledge of this area based on a lightweight literature review and consensus from domain experts. Accomplishing this may help provide a computable ontology artifact that can link health data to concepts involving SDoH and advance informatics methods and tools to understand the impact each determinant has on human populations. In addition, modeling SDoH may also help to reduce existing gaps in the literature through explicit links between the determinants and biological factors. This in turn can allow researchers and clinicians to make better sense of data and discover new knowledge through the use of semantic links.

Existing relevant ontologies usually focus on biology and biomedicine; however, the scope of SDoH expands beyond the biomedical level (ie, microlevel) and relates to aspects that are not necessarily biology based, such as economics, public policy, social factors, and so on. Some of the more mature ontologies, such as the ones hosted on the OBO Foundry, have some interoperability due to a shared framework, but there remains a need to connect the heterogeneous SDoH concepts within the biomedical level and elucidate meaning from the knowledge. We therefore put forth the following research objective: *using ontological methods, we can represent, formalize, and connect concepts pertaining to social, policy, and economic factors of SDoH.* The output of this effort is an initial ontology artifact that models the social, policy, and economic concepts and their relationships in composing the scope of SDoH to build future work. To accomplish this, we (1) analyzed the literature on the 3 aspects and the aforementioned concepts within these aspects and (2) produced an evaluated ontology artifact that reflects the intricate connections of the social and economic concepts of SDoH. This final experimental ontology artifact will be logically consistent with evaluation from domain experts and reasoning tools, grounded from a review of the literature to determine high-level concepts that stretch across SDoH, and aligned with a shared framework for biomedical ontologies to enable interoperability and reusability.

## Methods

### Overview

A brief yet comprehensive review was conducted to develop ontology terminology that effectively captures the concepts related to SDoH. This review served as a foundation for structuring and defining the key elements within the ontology. The literature reviewed aimed to examine how human health is affected by nonbiological factors that are associated with SDoH. The concepts were curated in concept map drafts from the review of SDoH, and the determinant of health model was used as a guide for concept development [13]. Later, we used Web Ontology Language, version 2 (OWL2) [14], the BFO [15-17], and semantic reasoners to construct and validate the ontology artifact.

### Review of Social and Economic Factors Impacting Health

Peer-reviewed articles were searched and evaluated by the primary author on PubMed from September 17 to October 8, 2021. Boolean operators and MeSH (Medical Subject Headings) terms were used to refine literature searches conducted using the advanced search feature on PubMed. Multiple concepts and relationships were combined through Boolean expressions, that is, “Social determinants of health AND (health policy OR health care system),” to broaden the search. Certain phrases were enclosed in parentheses to isolate parts of the search query for precision and specificity. MeSH terms with regard to SDoH were provided by PubMed and used to construct the queries. A summary of each search is described in Table 1.

**Table 1:** Literature search overview. Advanced search queries for each level of the social determinants of health were searched on the PubMed database between September 17 and October 8, 2021. The table displays the query, applied filter, and number of results each search yielded (N=2833).

Level	Search query	Results, n (%)
Macrolevel	“Social determinants of health AND (health policy OR health care system OR health disparities)”	721 (25.45)
Macrolevel	“Income inequality AND welfare AND health policy”	10 (0.35)
Macrolevel	“Environmental determinants of health AND climate change”	216 (7.62)
Mesolevel	“Work OR socioeconomic position AND (health inequalities)”	300 (10.59)
Mesolevel	“Socioeconomic outcomes AND (housing OR food)”	291 (10.27)
Mesolevel	Food OR poverty AND (health inequalities)”	250 (8.82)
Mesolevel	“Social determinants of health AND (crime rate OR domestic violence)”	14 (0.49)
Microlevel	“Social determinants of health (gender OR age OR ethnicity OR race OR inequalities OR education)”	1031 (36.39)

Articles of interest must have met the following criteria: free full text available, publication date <10 years ago, and published in English. With accessibility in mind, free full text was included as an eligibility criterion. Older publications may have been relevant to this paper but were not considered because they may not reflect current knowledge. Thus, the publication date was set to <10 years ago. As English is the primary language of all authors of this study, it was included as an eligibility criterion for the literature search. Finally, the article type must have been a book or document, systematic review, journal article, observational study, case report, or clinical study. Collectively, the search queries yielded a total of 2833 nonduplicate citations.

**Figure 1:**
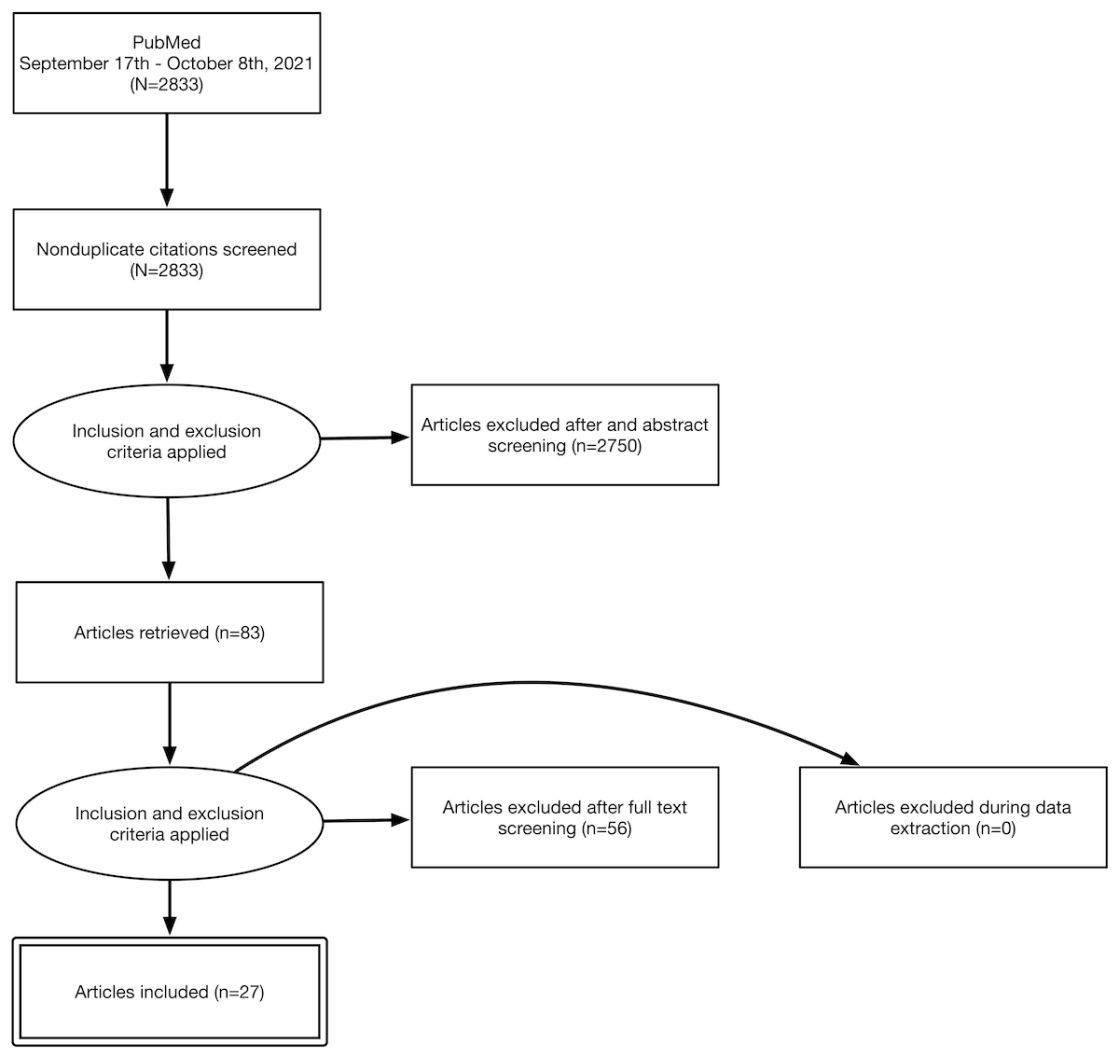
Iterative process for gathering the articles of interest. The PubMed searches produced 2833 nonduplicate citations; by applying the inclusion and exclusion criteria described in the main text, we removed 2805 (99.01%) citations, leaving 27 (0.95%) articles for review.

The first step of the inclusion and exclusion process involved removing articles that did not incorporate, or relate to, the MeSH terms identified in the search queries within their abstract or title. After this evaluation, of the 2833 articles, 2750 (97.07%) were immediately excluded, and 83 (2.93%) remained for a second assessment. Articles that did not precisely align with the research topic were once again removed. As a result, of the 83 articles, 56 (67%) were excluded, and thus 27 (33%) articles remained. Of these 28 articles, 9 (33%) were included for the macrolevel determinants, 10 (37%) addressed the mesolevel determinants, and 9 (33%) focused on the microlevel determinants. The inclusion and exclusion processes are depicted in Figure 1.

### Ontology Design and Development

**Figure 2:**
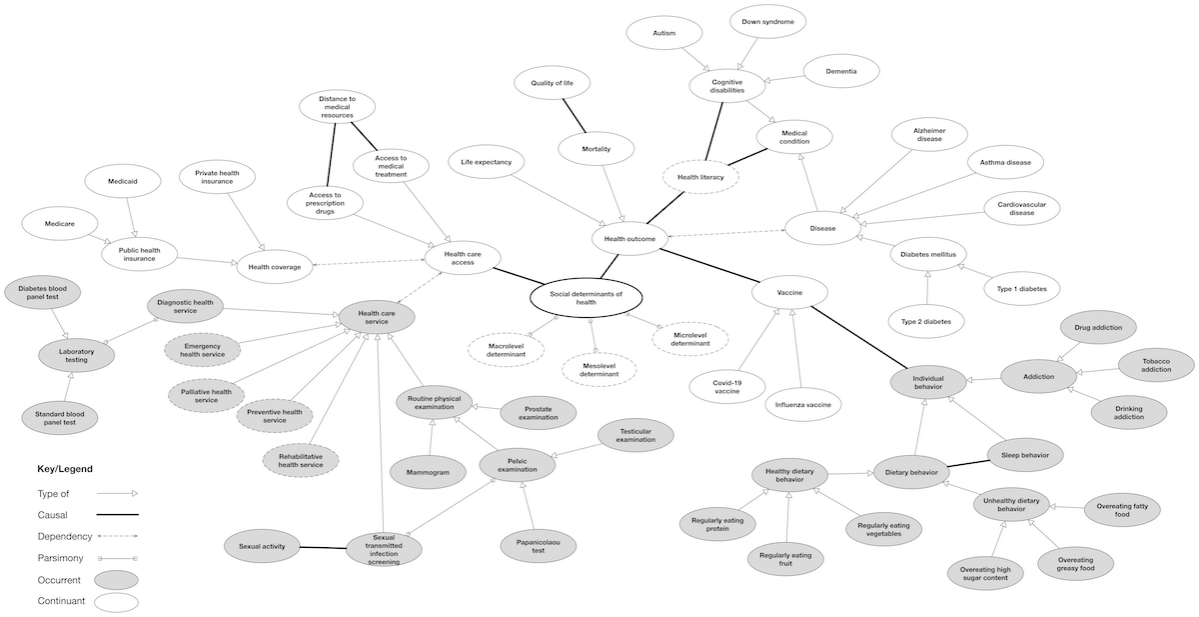
Determinants that impact health outcomes and behaviors. Dotted concept ovals indicate additional child concepts that are further described in Figures 3-5.

**Figure 3:**
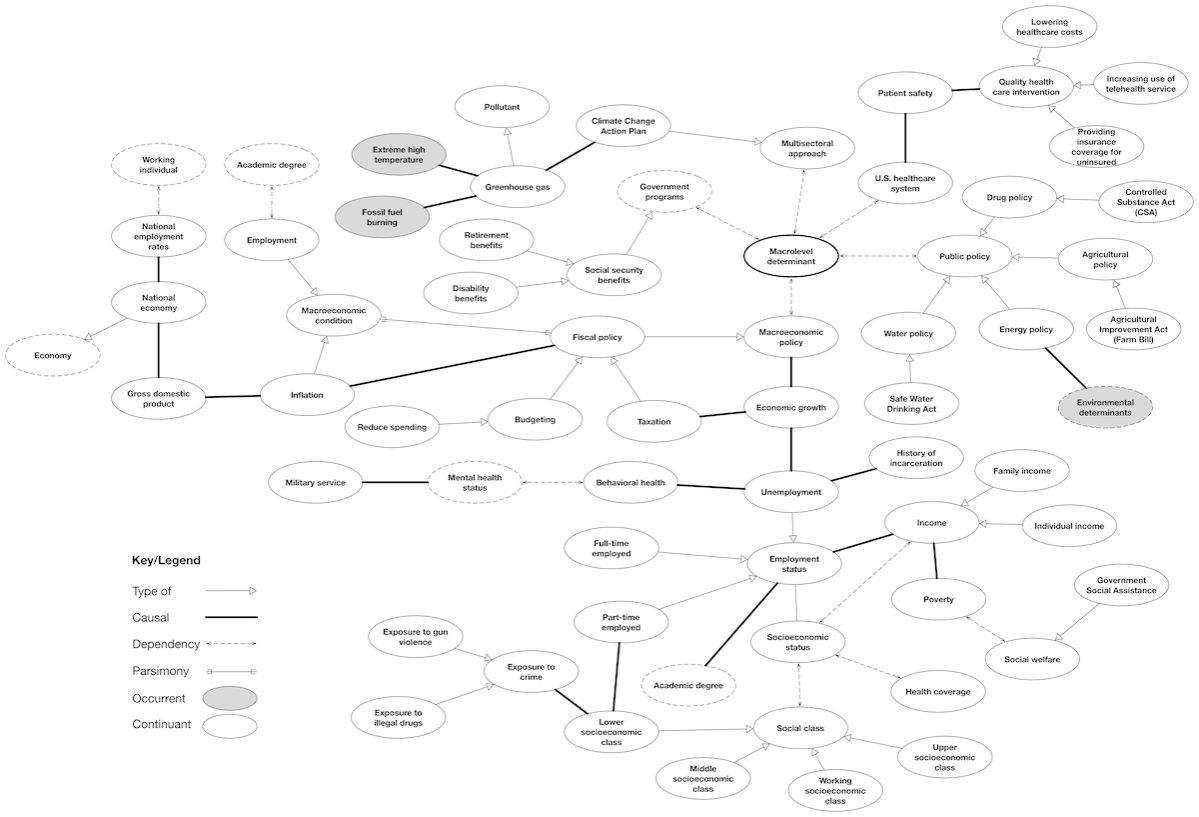
The relationship of concepts associated with macrolevel determinants. Concepts were derived from literature keywords, such as “health policy,” “income inequality,” “welfare,” and “environment.” Dotted concept ovals indicate additional child concepts.

**Figure 4:**
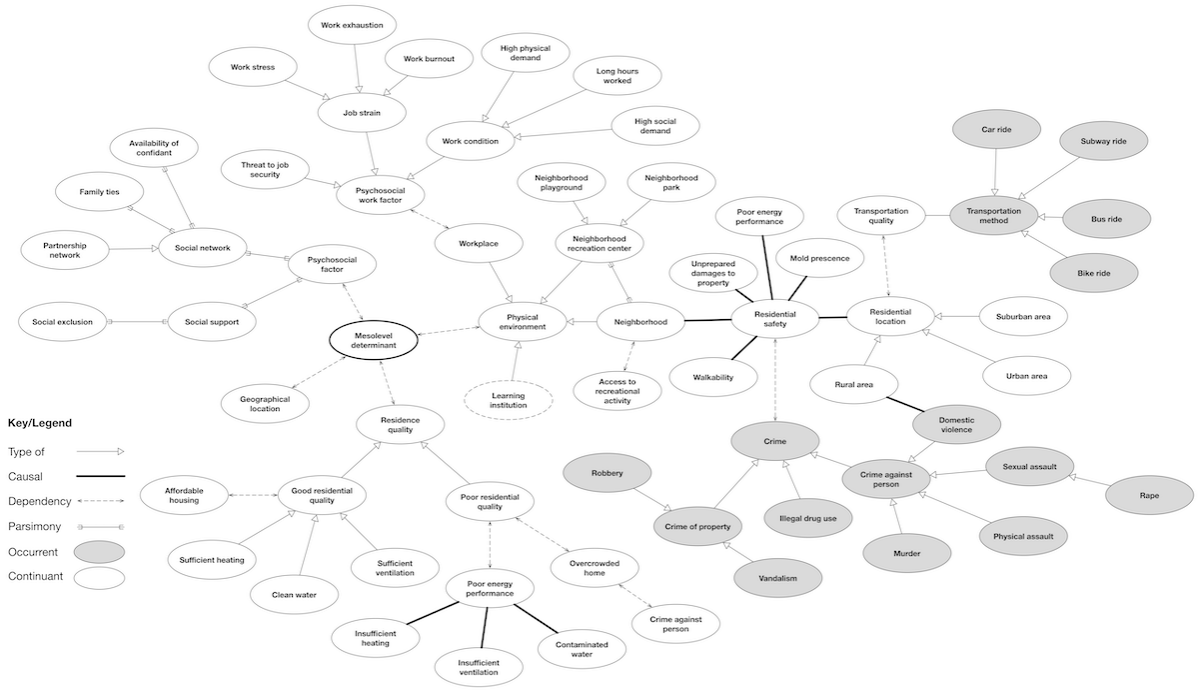
The relationship of concepts associated with mesolevel determinants. This map displays the most detailed network of relationships and was formed from the following keywords: “work,” “work conditions,” “psychosocial work factors,” “socioeconomic position,” “socioeconomic outcomes,” “food,” “poverty,” “housing,” and “crime.” Dotted concept ovals indicate additional child concepts.

**Figure 5:**
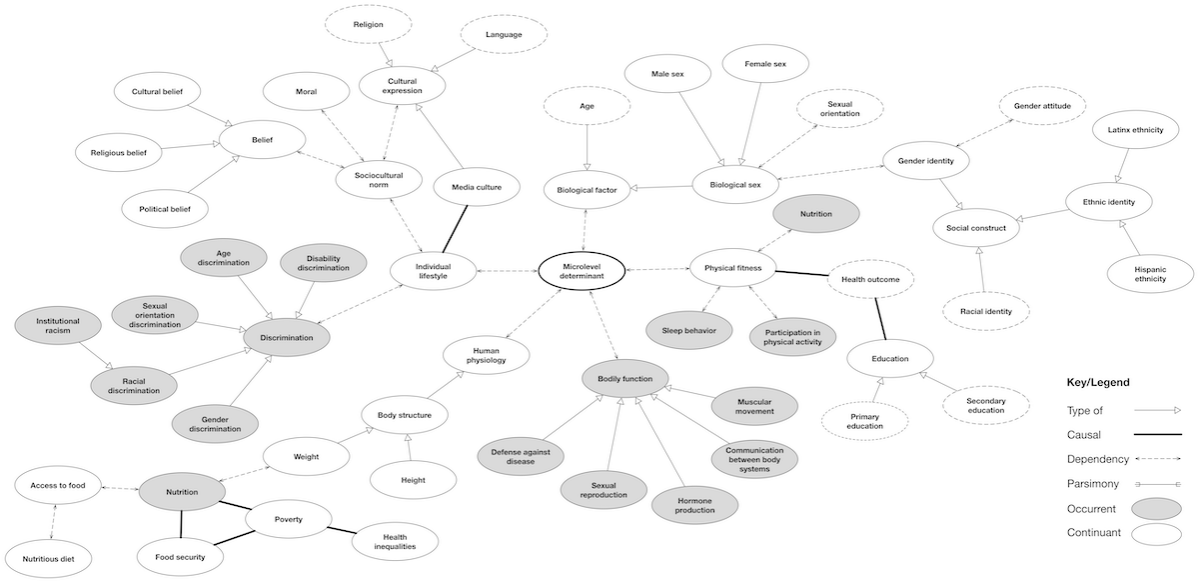
The relationship of concepts associated with microlevel determinants. Key elements of this map were gathered from keywords such as “physiology,” “gender,” “ethnicity,” “race,” and “behavior.” Dotted concept ovals indicate additional child concepts.

The review helped us capture some basic salient high-level knowledge that we can encode into ontology from concept maps. The motivation is to gain a *bird’s-eye view* of SDoH and proceed from a top-down approach in developing the experimental ontology. We developed iterative multiple concept maps using draw.io [18] to identify concepts and relationship links among the concepts. Our analysis of the concept maps revealed 4 generalized relationships that bridged the various concepts: *type of, part of, dependency,* and *causal*. Figures 2-5 reveal the final drafted concept maps.

The *part of* relationship is illustrated with a forked link connection and indicates that 2 concepts were part of each other; for example, this is demonstrated in Figure 2 between the concepts “Macrolevel determinants” and “Social determinants of health,” where macrolevel determinants are one part (meronym) of the composition of SDoH (holonym). A *dependency* relationship was demonstrated as a dotted-line link connection and referred to concepts that were dependent on each other. This can be seen in Figure 3 between the concepts “Behavioral health” and “Mental health status,” where an individual’s behavioral health is dependent on the status of their mental health.

A *causal* relationship was represented as a thick line link connection and described 2 concepts that had a cause-and-effect relationship. An example of this is demonstrated with the concepts “greenhouse gas” and “extreme high temperature,” where there is a causal relationship between greenhouse gas and increased temperatures. Finally, a *type of* relation was illustrated as an open arrowhead similar to Unified Modeling Language notation. This was used to represent inheritance, or parent and child concepts. All child objects inherit the functionality specified by the parent. This included relationship types; for example, as seen in Figure 2, the concept “Health outcome” was described to have a causal relationship with “Vaccine,” where “Vaccine” could have child entities such as “COVID-19 vaccine” and “Influenza vaccine.” As health has a causal relationship with vaccine, it also shares this relationship with its child objects.

### Ontology Encoding and Natural Language Evaluation

Items from the visualization concept modes were authored as an OWL2-based ontology [14] using the open-source ontology editor, Protégé [19]. Natural language translation was used to produce statements from the ontology for evaluation using Hootation, an experimental software library that extracts ontology axioms and produces human-friendly natural language statements for expert evaluation [20]. Statements were represented as sentences based on ontology class axioms and object properties; for instance, Hootation produced existential-type statements for subclass axioms such as “Every bus ride is a type of transportation method.” The evaluations were used to determine whether classes and their relationships were expressed correctly. To assess the quality of the ontology, 4 evaluators were asked to assess each statement on an Excel (Microsoft Corp) spreadsheet. Two of the evaluators are social behavioral scientists (ie, social work), and 1 of the evaluators is a biomedical ontology scientist. Furthermore, each evaluator was asked to rate a statement as (1) “Yes, this is accurate,” (2) “No, this is not accurate,” or (3) “Do not know if this is accurate.” The Results section discusses details of our analysis from the collected evaluation data.

### Alignment With the BFO

To ensure semantic interoperability, we aligned our ontology with the BFO [15,17,21]. The BFO is an upper-level ontology that models entities using metalevel categories based on philosophical realism [16]. It is a widely regarded standard framework for creating biomedical and health reference ontologies that enable sharing, interoperability, and consistency with other ontologies by way of the metalevel categories and properties. To advance this work further, we aligned our exploratory ontology with a few of the metalevel concepts from the BFO. Currently, we have made some early attempts to align the object properties with OBO Foundry properties.

Earlier, we identified 4 basic relationships that connect the concepts from our ontology model. We reviewed the BFO model and identified object property relationships that semantically correspond with our 4 relationship connections. The OBO Foundry’s p*art of* or *has part* (BFO_0000050 [22]) object property was used to reflect the *part of* relationship [23]. The OBO Foundry’s *causally related to* (RO_0002410 [24]) object property reflected a causal relationship, and the OBO Foundry *depends on* (RO_0002502 [25]) object property was used to reflect the dependency relationship [23]. Naturally, the type of relationship was handled by OWL2’s *SubClassOf* axiom.

In addition to the identified property relationships, we settled on classifying the concepts using the 2 basic categories *continuant* (BFO_0000002 [26]) and *occurrent* (BFO_0000003 [27]). A *continuant* is defined as “an entity that persists, endures, or continues to exist through time while maintaining its identity” [26], essentially an entity or object. An *occurrent* is defined as “an entity that unfolds itself in time or it is the instantaneous boundary of such an entity (for example a beginning or an ending) or it is a temporal or spatiotemporal region which such an entity occupies_temporal_region or occupies_spatiotemporal_region” [27], basically an event or process. Each of the concepts in our model was classified into these 2 very basic classes from the BFO. Classifying these concepts into these BFO categories helped leverage the aforementioned property relationships because they were dependent on whether the connecting concepts were aligned with the BFO concepts.

We used ROBOT (a recursive acronym for “ROBOT is an OBO Tool”) [28], an OBO Foundry command line software tool, to perform development tasks with OBO Foundry ontologies. We extracted the 2 BFO categories, and the 3 object properties (along with their corresponding axioms via the STAR [situation, task, action, and result] method) using ROBOT to generate a light import of the essential BFO terms. The exported import was used to encode alignment of the concepts in our ontology with the BFO, and FaCT++ [4] was the software reasoner of choice, due to its fast performance, to test whether our ontology model was logically satisfiable. The finalized reviewed ontology, named 3M (microlevel, mesolevel, and macrolevel) Ontology, was published in our GitHub repository [29].

## Results

### Overview

The literature search identified several topics of discussion for each determinant level. For macrolevel determinants, topics included health policy, income inequality, welfare, and the environment. For mesolevel determinants, the selected articles investigated areas such as work, work conditions, psychosocial work factors, socioeconomic position (SEP), socioeconomic outcomes, food, poverty, housing, and crime. Among all 3 levels, the highest number of articles for discussion were available for mesolevel determinants. Finally, the articles found for microlevel determinants examined gender, ethnicity, race, and behavior. In the following paragraphs, we discuss SDoH in detail.

### Policy Making

Social policies and programs, fair employment and working conditions, and living environment are all likely to have the greatest impact on SDoH [30]. Social protection measures, increased coverage and quality of early years care, parental employment support, and gender equality in employment and education may improve early childhood development and even help to reduce child poverty. Affordable housing can be met through minimum housing standards and government actions [30]. Air quality legislation may have some benefits on air pollution and overall living [30].

The effects of climate change may be reduced by improving early warning systems and extreme weather preparedness. Without action, climate change has the potential to raise agricultural prices, and this may threaten food security in low-income regions [31]. Families and individuals with low-income status are most susceptible to climate-related diseases such as malaria. Providing universal health care coupled with climate resilience measures is needed to reduce climate change impact on those with low-income status [31]. Bouzid et al [32] point out that several systematic reviews discuss diseases associated with climate change, but more focus should go toward the management of droughts, floods, air pollution, and food safety. The lack of research in these areas is likely due to the unpredictable nature of, for example, floods and government bodies that are primarily concerned with disaster response rather than research [32].

### Policy Outcomes and Interventions

Health policies are fundamental for health and safety and are designed to improve quality of life. The most common types of implementation measures used to assess health policy outcomes include acceptability, feasibility, appropriateness, and compliance [33]. Well-tested quantitative measures are not used enough, and this may directly affect policy outcomes [33]. Most policy intervention tools at the school, district, state, or province level assess wellness policies from high-income countries such as the United States. Data from a systematic review showed that low- and middle-income countries lacked policy intervention initiatives [34]. Similar studies have investigated the relationship between income inequality and subjective well-being.

Evidence on the impact of social assistance on human health remains unclear [35]. Not enough articles discuss the differences between social assistance recipients and nonrecipients [35]. There is a lack of strong methods and study designs to evaluate the health effects of policies mainly in part due to insufficient data. Population-based health surveys do not provide enough information on respondent characteristics [35]. The available methods used to evaluate policy interventions require researchers to identify instances of large-scale policy change when social assistance programs are hardly ever affected by big changes. Instead, areas to be looked at are tobacco, food labeling, greater income redistribution, and labor market regulations [35].

A systematic review assessed randomized social experiments on social policy interventions for health outcomes in the United States and found that investments in early life, income support, and health insurance interventions may hold the potential to improve mental health and health in general [36]. The authors’ power analyses suggested that the models that were used were underpowered to detect health effects and outcomes. The authors noted that policy-related experiments should focus on design to accurately measure the relationship between health outcomes and policy interventions.

### Income Inequality and Low SEP

According to a meta-analysis, income inequality was not influenced by measures used to assess subjective well-being or geographic region [37]. Instead, the level of country development, more specifically job opportunities, may be linked to income inequality in low- and middle-income countries. This serves as an indicator to government policy makers that reducing income inequality may lead to an improvement in subjective well-being [37]. While income inequality may have some effect on well-being, political economy may also influence population health. A systematic review revealed that there is a gap in the literature on many aspects of political economy, and it is unclear whether there is a relationship between political economy and population health [38]. Although there is no evidence, it seems that social democratic states with higher public spending tend to have better population health, but there is still no significant relationship between welfare state type and health inequalities [38].

In addition to income inequality, a low SEP may also contribute to poor health outcomes. There is consistent evidence that individuals who have a low SEP are often associated with hospital death and poor-quality end-of-life care [39]. Individuals with a poor education and who resided in impoverished neighborhoods were most likely to die in the hospital, receive acute-based care, and not receive specialized palliative care [39]. Future research on end-of-life interventions should consider SEP and its effects across the social strata [39].

### Physical Environment and Health

A systematic review conducted by Lago et al [40] analyzed the relationship between health and physical environment, lifestyle, and social and economic conditions. On the basis of their evidence, the authors concluded that the main factor linking socioeconomic status and health status was income. Individuals with a higher level of income, as opposed to those with lower income, were associated with a lower chance of negative health outcomes [40]. The current association between income distribution and health is the general conclusion because individuals belonging to a lower social class have been shown to have worse average health. Different variables such as education may also play a role in determining health status because it is usually correlated to individuals’ social class [40]. Warmth and energy interventions may lead to improvements in respiratory health, mental health, and overall health for individuals with low-income status. Studies that targeted existing chronic respiratory diseases linked to inadequate warmth were most likely to see health improvement [41].

A mixed methods study demonstrated that energy performance interventions reduced energy use and helped raise indoor temperatures [42]. Despite there being a lack of evidence that suggests that energy performance investments improve health, data did show that improvements in social and economic conditions are better for overall well-being and health [42]. Economic conditions such as a low SEP are linked to poor health outcomes [42]. Individuals with a low SEP had an increased risk of cardiometabolic disorders and mortality according to Petrovic et al [43], who examined the role of health behaviors in socioeconomic equality in health. Behaviors such as smoking, alcohol consumption, physical activity, and diet were considered, as well as health outcomes such as cardiometabolic disorders and mortality. Of all behaviors examined, smoking contributed to the most social inequalities in health. The authors conclude that health behaviors may contribute to socioeconomic inequalities, but this is dependent on population and study characteristics [43].

### Impact of Food Availability on Nutrition

Individuals with low- and middle-income status are subject to food scarcity and poor nutritional health [44]. Supplementary feeding had a positive effect on weight and growth in low- and middle-income countries and was most beneficial to individuals who were poorly nourished. There were moderate positive effects on psychomotor development and mixed evidence on improved cognitive development [44]. Groups with lower income tend to select energy-dense diets that do not consist of vegetables or fruit [45]. Fats, refined grains, and added sugars are less expensive than nutrient-dense foods [45]. As a result, there may be a link between high obesity rates and low-cost calories [45]. Pregnant or postnatal women had an increased intake of fruits and vegetables after being enrolled in a food subsidy program [46]. Mean birth weight was slightly higher in 2 high-quality studies [46]. There is currently not enough evidence on the true impact of food subsidy programs for both children and adults [46].

### Work Conditions and Occupational Health

Currently, no data suggest that workplace health promotion programs (WHPPs) increase socioeconomic inequalities in health, and there is not enough quantitative data on the ability of WHPPs to reduce social inequalities [47]. WHPPs seem to be the most helpful for working individuals who have a low SEP, but most of the programs were equally effective for groups from lower and higher socioeconomic backgrounds [47]. Most studies on working conditions supported the notion that adverse working conditions can mediate the association between SEP and well-being [48]. Studies that examined occupational categories or employment grades as indicators of SEP had the strongest findings in comparison to those that used education or income [48].

There is strong evidence that both physical and psychosocial factors are the cause of approximately one-third of the socioeconomic inequalities in health [49]. Despite limited longitudinal studies, cross-sectional evidence consistently showed that both physical and psychosocial work factors contributed to socioeconomic differences in self-rated health. Work factors may also play a role in inequalities, but there is not enough evidence to determine specific types of work factors [49]. In comparison to men, women experienced worse working conditions and higher job insecurity and also experienced poorer self-perceived physical and mental health [49,50]. Employed men had less emotional support, worked longer hours, and faced higher physical demands; however, they also held higher job statuses and had greater levels of effort-reward imbalance [50]. Although men were subject to more physically demanding tasks, women reported more musculoskeletal symptoms [50]. Health disparities between genders may stem from less favorable working conditions experienced by women [50]. Women are more commonly exposed to repetitive movements with low loads and awkward working positions than men [50]. Anthropometric differences in bone mass, fatty tissue, and muscle may also influence these health outcomes [50].

### Socioeconomic Factors and Domestic Violence

Employment, income, social class, ethnicity, race, and living conditions all make up socioeconomic factors that may contribute to domestic violence [51]. The highest frequency of violence against women is found in a family environment, with the spouse being the most common perpetrator, and is most prevalent in low-income countries [51]. Individuals who experienced sexual dissatisfaction, unsatisfactory environmental conditions, and mental disorders tend to partake in acts of violence [51]. Certain countries have established laws to better protect women, but there needs to be an integrated approach for both national and international government organizations to achieve social change [51].

### Discrimination and Poor Health Outcomes

The literature has shown a significant relationship between poor health and racism and a relationship with even higher significance between poor mental health and poor physical health [52]. Health outcomes indicated an association between racism and suicidal ideation, planning, and attempts. Depression was the most reported health outcome and had the same magnitude of association as racism [52]. Health care providers with different training, experience, and specialty backgrounds may hold implicit bias against racial and ethnic minority people [53]. A systematic review revealed that bias is associated with patient-provider interactions rather than health outcomes [53]. This indicates that patient-provider interaction can mediate the relationship between provider bias and patient health outcomes [53].

Institutionalized racism refers to the macrolevel systems, social forces, institutions, ideologies, and processes that interact with one another to cause inequities among racial or ethnic groups [54]. Although public health literature mentions the term *institutionalized racism*, it does not always engage with the concept or dive deep into the mechanisms through which health injustice is perpetuated [54]. To better understand racial and ethnic groups considered disadvantaged, the term should be explicitly mentioned in public health research as a central concept of health inequities [54]. Disparities in the neonatal intensive care unit exist in structure, process, and outcomes and generally disadvantage infants from racial and ethnic minority groups [55]. Hispanic and Black infants are most likely to receive care in poor-quality hospitals. In addition, hospitals serving racial and ethnic minority groups are underresourced and may lack quality improvement infrastructure. Quality improvement initiatives may have the best effect on populations considered disadvantaged who experience poor-quality care [55].

### Gender Attitude and Sociocultural Norm

There may be several factors that can shape gender attitudes in early adolescence. In a study conducted in 29 countries, data demonstrated that young adolescents from varying cultures all express similar stereotypes and gender attitudes [56]. A gender study demonstrated that adolescents commonly endorsed norms that perpetuated gender inequalities such as masculinity established on toughness and skills or femininity based on physical appearance and shaming of sexuality [56]. Sociodemographic characteristics such as gender, race, immigration status, and age cause a variation in the results; however, family and peers are the central influences in building gender attitudes [56].

### Statistical Analysis

Initial metrics from the ontology exhibited 245 classes, 47 object properties, and 346 logical axioms. Four evaluators independently reviewed 232 statements, specifically SubClassOf axioms, produced by the Hootation natural language translation software. Each statement was categorized as a *0* or a *1* to indicate expression accuracy. Statements that were not accurate were annotated as *0*, and accurate statements were annotated as *1*. Unsure responses were annotated as *0*. The levels of agreement for each evaluator were calculated using a web-based program called ReCal3 (“Reliability Calculator for 3 or more coders”) [57]. Intercoder reliability was assessed through an average pairwise agreement and an average pairwise Cohen κ value [58].

Individual levels of agreement were as follows: evaluator 1=54%, evaluator 2=58%, evaluator 3=56%, and evaluator 4=76%. The average percentage agreement in terms of the average number of shared responses was 60.85% (SD 10.13%). The pairwise agreement also demonstrated that evaluator 2 and evaluator 4 had the highest similarity (74.14%) among shared responses, and the lowest percentage for shared responses was between evaluator 1 and evaluator 3 (48.71%). The pairwise agreements between evaluator 1 and 2 (56.47%), evaluator 1 and 4 (70.26%), evaluator 2 and 3 (60.35%), and evaluator 3 and 4 (55.17%) were recorded. The relationship among these results is demonstrated more accurately through the average pairwise Cohen κ value (0.19), which determined the interrater reliability. The results are presented in Table 2.

**Table 2:** Evaluation of subclass accuracy in percentage. Evaluators were asked to rate expression accuracy with 0 (no and unsure) and 1 (yes). The individual levels of agreement and disagreement are shown for each evaluator.

	Agreed (*yes*^a^; %)	Disagreed (*no*^a^ and *unsure*; %)
Evaluator 1	54	46
Evaluator 2	58	42
Evaluator 3	56	44
Evaluator 4	76	24

^a^*Yes* indicates the evaluator denoted a knowledge statement from the ontology was true, whereas, *no* indicates the evaluator assessed it to be false and unsure for if the statement was unknown to the evaluator to be true or false.

The average Cohen κ value was extremely low (0.19), as was the pairwise Cohen κ value for evaluators 1 and 3 (−0.04). The other Cohen κ values between evaluators 1 and 2 (0.12), evaluators 1 and 4 (0.38), evaluators 2 and 3 (0.19), evaluators 2 and 4 (0.44), evaluators 3 and 4 (0.04) were recorded. The statistical analyses helped identify concepts that required revision or omission. Statements that were classified as *0* were reviewed for analysis and possible error. Concepts with high levels of disagreement were revised, and new concepts were added to create a more logically structured ontology.

## Discussion

### Principal Findings

The ontology that was developed attempted to model the macrolevel and mesolevel conceptualizations of SDoH in more detail. Interpretations from the literature demonstrated that macrolevel factors are crucial determinants of health and health inequities. Individuals considered disadvantaged are almost always at risk for poor health and poor health outcomes. The main drivers of health inequalities seem to be a lack of education, affordable housing, basic housing needs, income, and access to health care. More specifically, women and racial and ethnic minority people are subject to these determinants, and this is the same for individuals living in low- and middle-income countries. Data from the articles also identified gaps in the literature for current research on low- and middle-income societies. Moreover, policy outcomes determined by SDoH can be measured in many ways; yet, there is little quantitative data on their validity. Finally, findings from the literature provided a solid foundation of knowledge and analysis that guided the design and development of the ontology.

Each of the 3 determinant levels interacts with, and dynamically influences, the other 2; therefore, delineation among the micro-, meso-, and macrolevel determinants is not always clear [59]; for example, the primary effects of discrimination are microlevel factors, such as the imposed psychological context from the individual enacting the discrimination and the individual experiencing it. However, the act of discrimination also has effects on the meso- and macrolevel determinants. The willingness of providers to live and work in underserved communities is considered a mesolevel factor, while the ability of the health care system to create recruitment and retention policies is a macrolevel factor. For an adequate transformation of these complex systems to occur, there will need to be an emphasis on the interactions among the levels and their interdependence [60]. Our work is imperative to the understanding of the ontology of SDoH because it will further the scholarly understanding of public health, lead to the development of necessary policy and interventional changes, and reduce the gap in health care literacy.

The statistical analyses from the evaluations were used to create a revised version of the ontology with a broad spectrum of knowledge concepts ranging from the macrolevel to microlevel determinants. Interpretation of the statements varied, and this may have posed a potential challenge for proper ontology evaluation; for example, the average Cohen κ values indicated that there was no effective agreement, implying that statements from the ontology were not accurate. The low levels of agreement were mostly attributed to poor labeling and poor association between class and subclass axioms. Poor labeling referred to items that were not specific enough (eg, burnout→job strain, translated to “every burnout is a job strain”). Poor associations among expressions were found to be untrue or mislabeled (eg, poor→income inequality, translated to “every poor is an income inequality”). Personal opinions on statement evaluations were considered but not always incorporated for revision; for example, the concept poor energy performance was not understood by the first 3 evaluators, but it was cited in the literature and described poor energy efficiency in homes, such as poor heating or poor insulation [42].

**Figure 6:**
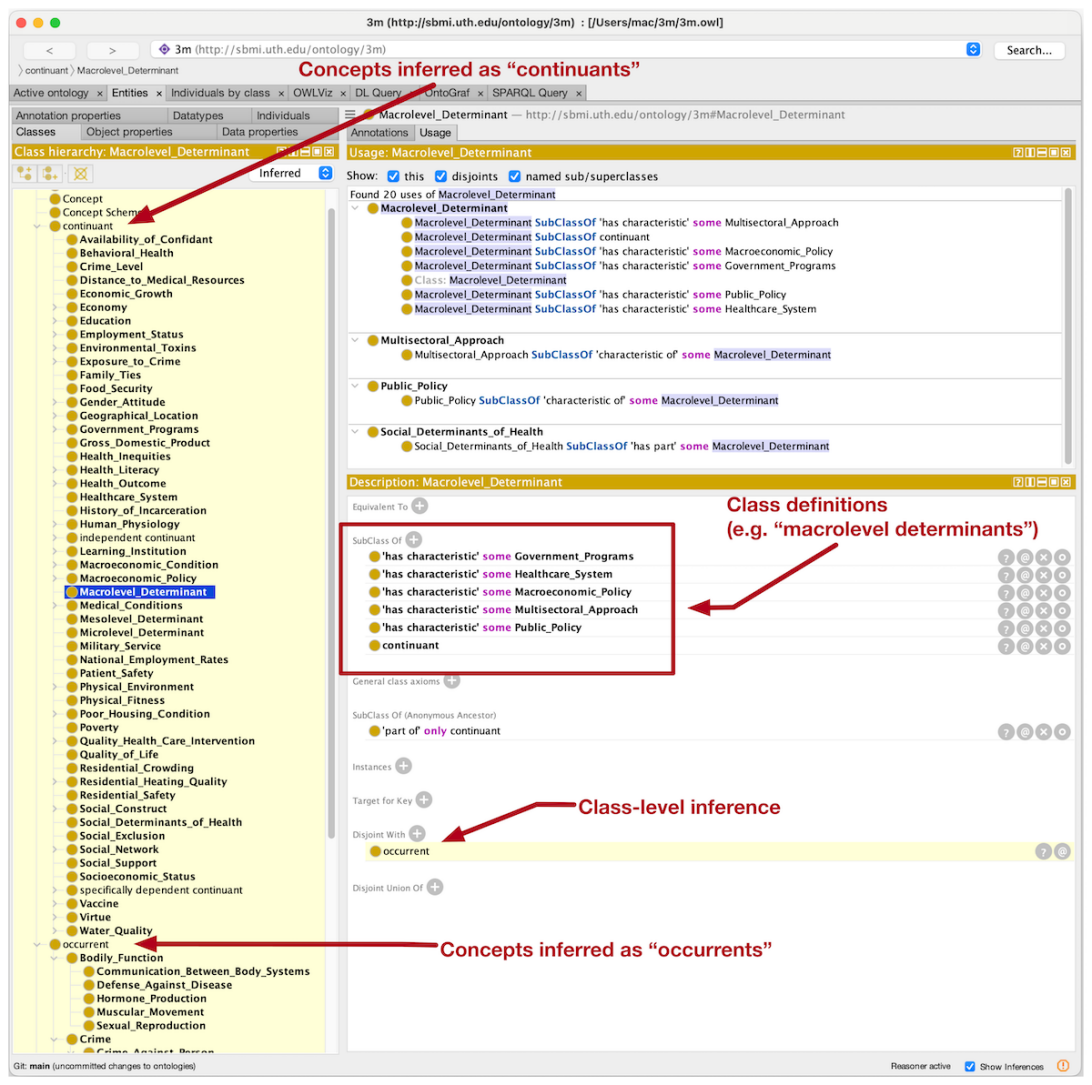
Screenshot of the experimental ontology in Protégé with alignment with Basic Formal Ontology concepts and properties.

After some iterative revisions of the ontology, we imported the minimal BFO concepts and property relationships discussed earlier into the Protégé environment and encoded the concept alignment with the BFO terms. We used the FaCT++ reasoner to perform a check of the logical consistency of our final aligned ontology model, and it revealed no inconsistent axioms. At the time of this writing, the core ontology exhibited 383 classes, 109 object properties, and 748 logical axioms, and we included an import of the Simple Knowledge Organization System ontology for additional annotation properties [61]. This preliminary ontology is currently hosted on GitHub [62]. Figure 6 shows a screenshot of the ontology in the Protégé tool with all essential concepts aligned (by assertion and inference) with the BFO categories and properties.

Determining the accuracy of ontology concepts may help to produce a well-structured ontology. Moreover, appropriately addressing SDoH is fundamental for improving health and reducing long-standing inequalities. Modeling concepts transform metadata into a knowledge domain, which facilitates new knowledge discovery. By linking this ontology of SDoH with other biomedical ontologies, researchers can make use of shared data for data exchange and information integration for biomedical tools such as computer-aided reasoning or decision support applications, enhance existing ontology knowledge bases, produce precise definitions of SDoH concepts in natural language, and provide a better understanding of the terminology associated with SDoH to reduce gaps in the literature.

Several concepts exist beyond the macro-, meso-, and microlevel determinants, which are included in the final version of the ontology. Concepts that impact or contribute to SDoH include academic degree, access to food, access to health care, behavioral health, discrimination, distance to medical resources, economic growth, economy, employment status, environmental determinants, exposure to crime, disease, food security, gross domestic product, gender attitude, gender identity, health inequities, health literacy, health outcomes, health services, health care coverage, history of incarceration, income, individual behavior, media culture, medical conditions, military service, national employment rates, nutritious diet, patient engagement, patient safety, personal health management, quality health care interventions, quality of life, sexual activity, sexual orientation, social class, social constructs, and vaccine. Each of these items contains additional subclasses (Figures 2-5).

### Macrolevel Determinants

Class axioms for the macrolevel determinants included government programs, health care system, income inequality, macroeconomic conditions, macroeconomic policies, multisectoral approach, public policy, social security benefits, and social welfare. Each of these classes has been broken down further, as illustrated in Figure 3. Government programs, social security benefits, and social welfare were created to assist individuals who belong to a low social class, have a secondary-level education or less, and who are unemployed or work minimum wage jobs [30]. Both national and local governments intend to improve overall health by formulating macroeconomic policies and implementing multisectoral action initiatives to develop comprehensive strategies for addressing SDoH, promote inclusion and transparency in decision-making, and adopt equity-focused approaches in planning and resource allocation [30].

Currently, the US federal government mandates several public policies to improve the quality of life through the drug policy, agricultural policy, water policy, and energy policy [12]. Macroeconomic conditions such as employment and inflation can help regulate the economy, but these are highly dependent on current national employment rates [38]. Likewise, fiscal policies may help to reduce government spending, control debt, and regulate taxation, which in turn controls the economy [38]. Findings from the literature are reported on adult populations and rarely focused on children.

### Mesolevel Determinants

The focal point for the mesolevel determinants is the physical environment. It is the level that contained the highest number of classes and subclass axioms. In addition to physical environment, classes included access to recreational activity, affordable housing, crime level, geographic location, psychosocial factor, psychosocial work factor, residence quality, residential location, residential safety, transportation, transportation quality, and walkability. The concepts that warrant the most discussion are physical environment, residence quality, and psychosocial work factor. The environment in which an individual lives and works affects their ability to function and socialize. The quality of housing has major implications on health outcomes [41]. Evidence suggests that poverty and low income affect housing circumstances.

Poor residence quality, such as insufficient heating or insufficient ventilation, may lead to illness [42]. Likewise, poor housing conditions such as mold presence, overcrowding, and unrepaired damage to property may also affect healthy living. Negative health outcomes are associated not only with residence quality but also with work environment. Exposure to psychosocial work factors was linked to poor mental health status [49]. Working long hours and being subject to high physical demands can result in depression, burnout, or work exhaustion [49]. Undesirable working conditions may affect job performance and ultimately employment status [48]. Occupations differ in both psychosocial work factors and work conditions; therefore, these concepts could be elaborated further. Mesolevel factors are presented in Figure 4.

### Microlevel Determinants

Microlevel determinant class axioms were identified as biological factor, bodily function, human physiology, individual factor, individual lifestyle, nutrition, participation in physical activity, and physical fitness. Each of these concepts has subclasses that are illustrated in Figure 5. The relationship between individual factors such as education and health is complex. Low educational attainment may result in poor health. Cognitive disabilities and health conditions may affect educational outcomes, which in turn affect health literacy [63]. Low health literacy is associated with poor health outcomes and mortality. Individuals who do not understand the severity of their health conditions are less likely to seek medical care [63]. Poor operation of bodily functions may also result in undesired health outcomes. Likewise, poor management of diet and nutrition can affect physical fitness [45]. Individuals with a low SEP are subject to food insecurity and often malnourished [44]. Their inability to purchase food or healthy food options reflects their diet and nutritional status, resulting in illness [44].

Another microlevel factor that disrupts healthy living is discrimination. Individuals who are discriminated against for their race, gender, sexual orientation, disability, or age may experience depression and suicidal ideation. Discrimination that occurs in a hospital setting is prominent against African American and Hispanic individuals and results in poor or delayed treatment [53]. Negative gender attitudes may elicit aggressive behavior and lead to domestic or physical violence [56]. Attitudes toward gender may be attributed to sociocultural norms or individual beliefs; for example, individuals living in low- and middle-income countries with a high poverty rate often express toxic masculinity [56].

Individuals who identify as lesbian, gay, bisexual, transgender, or queer are targets for discrimination, bullying, isolation, and violence [64]. This is true even in the health care system, where transgender women are commonly admitted as men, despite them expressing their gender [64]. Similarly, the normalized societal attitude toward individuals with disabilities is often exclusionary [64]. As health systems are often not designed with the needs of individuals with disabilities in mind, these individuals frequently face challenges, requiring them to navigate and challenge established norms [64]. Overall, findings from the literature emphasized that microlevel factors play a large role in human behavior and health outcomes.

### Conclusions

In this paper, we examined the range of social and economic factors covering SDoH and modeled these aspects using ontology-based methods and tools to create a representational artifact. With this artifact, data and resources can be linked and aggregated to address clinical research that could analyze the link between the aforementioned factors and possible biological factors sourced in published bioinformatics ontologies. To our knowledge, this is the first ontology to focus on knowledge concepts that are not addressed by current biomedical ontologies for SDoH. The latest version of this ontology is available on GitHub [62] for public early release and future updates. Overall, this preliminary work is a demonstration of the possibility to model these heterogeneous social and economic concepts that can be aligned with the greater body of biomedical ontologies.

However, the social and economic scope of SDoH is expansive, and although the ontology is broad, it is still in its early stages and could be expanded further with more granular social and economic concepts. Future consideration will be given to developing specific subdomains that can act as federated modules that can integrate with this ontology. Finally, we will include further aligning of this work with the BFO, using more precise semantic properties to accurately reflect the relationships among the concepts, which will provide further alignment with the existing validated biomedical ontologies.

## Abbreviations

BFO: Basic Formal Ontology
MeSH: Medical Subject Headings
OWL2: Web Ontology Language, version 2
SDoH: social determinants of health
SEP: socioeconomic position
STAR: situation, task, action, and result
WHPP: workplace health promotion program

## Data Availability

The data sets generated during and analyzed during this study are available in the 3M Ontology repository [29].
